# Fast LC-ESI-MS/MS analysis and influence of sampling conditions for gut metabolites in plasma and serum

**DOI:** 10.1038/s41598-019-48876-w

**Published:** 2019-08-26

**Authors:** Tom van der Laan, Tim Kloots, Marian Beekman, Alida Kindt, Anne-Charlotte Dubbelman, Amy Harms, Cornelia M. van Duijn, P. Eline Slagboom, Thomas Hankemeier

**Affiliations:** 1 Analytical Biosciences and Metabolomics, Division of Systems Biomedicine and Pharmacology, Leiden Academic Center for Drug Research, Leiden University, Leiden, 2333 CC The Netherlands; 2 BioMedical Metabolomics Facility Leiden, Leiden University, Leiden, 2333 CC The Netherlands; 3 Section of Molecular Epidemiology, Leiden University Medical Center, Leiden, 2333 ZA The Netherlands; 4 Department of Epidemiology, Erasmus University Medical Centre, Rotterdam, 3015 GE The Netherlands

**Keywords:** Cardiovascular diseases, Laboratory techniques and procedures, Nutrition, Biomarkers

## Abstract

In the past few years, the gut microbiome has been shown to play an important role in various disorders including in particular cardiovascular diseases. Especially the metabolite trimethylamine-N-oxide (TMAO), which is produced by gut microbial metabolism, has repeatedly been associated with an increased risk for cardiovascular events. Here we report a fast liquid chromatography tandem mass spectrometry (LC-MS/MS) method that can analyze the five most important gut metabolites with regards to TMAO in three minutes. Fast liquid chromatography is unconventionally used in this method as an on-line cleanup step to remove the most important ion suppressors leaving the gut metabolites in a cleaned flow through fraction, also known as negative chromatography. We compared different blood matrix types to recommend best sampling practices and found citrated plasma samples demonstrated lower concentrations for all analytes and choline concentrations were significantly higher in serum samples. We demonstrated the applicability of our method by investigating the effect of a standardized liquid meal (SLM) after overnight fasting of 25 healthy individuals on the gut metabolite levels. The SLM did not significantly change the levels of gut metabolites in serum.

## Introduction

**Figure 1:**
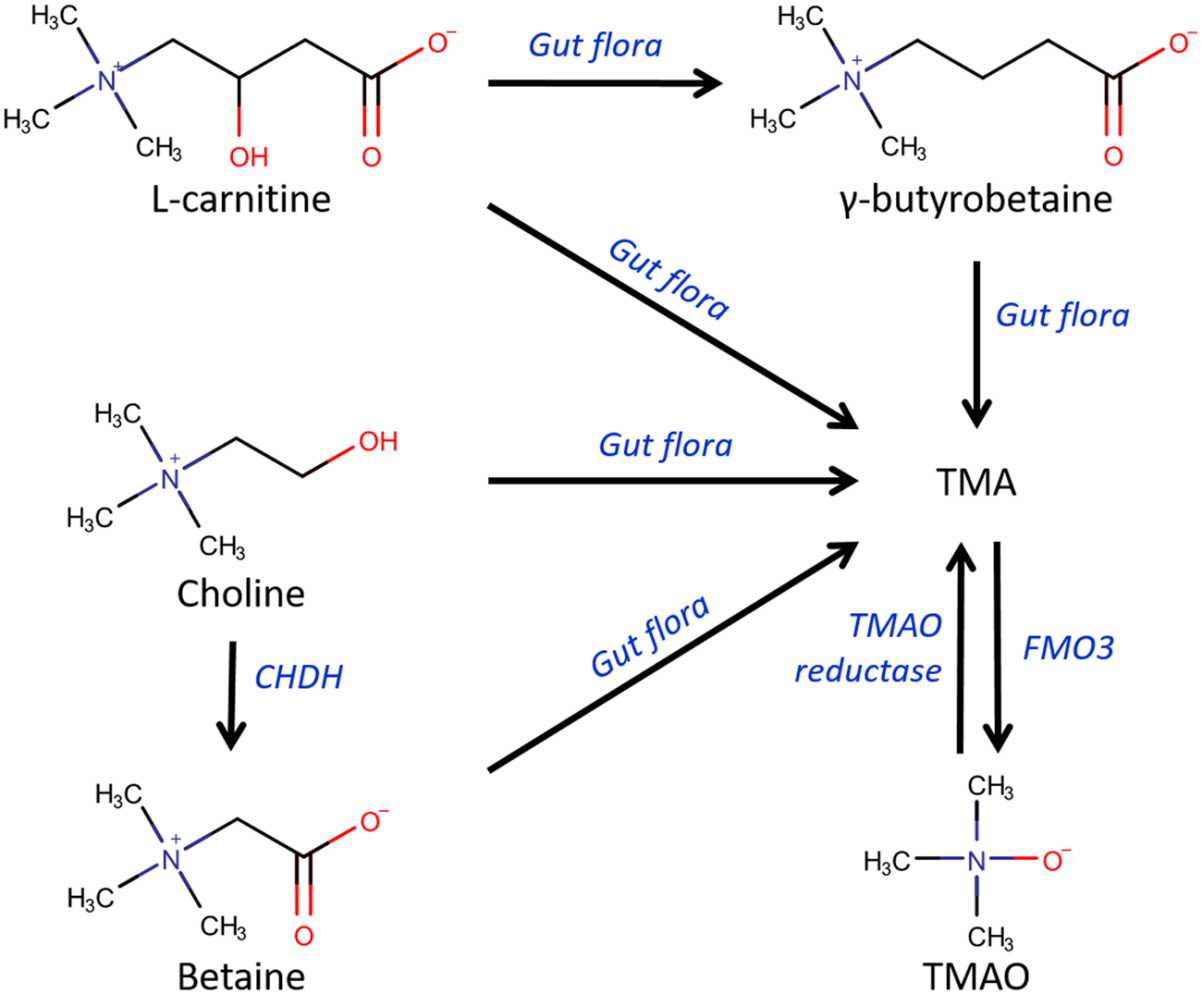
TMAO biosynthesis. L-carnitine and choline are derived from the diet and are endogenously synthesized. L-carnitine can be converted by the gut flora to TMA directly or with γ-butyrobetaine as an intermediate. Choline is converted to betaine by choline dehydrogenase (CHDH), with highest expression in the renal cortex. Betaine, as well as choline are a substrate for the gut flora to form TMA. TMA is converted to TMAO by flavin-containing monooxygenase 3 (FMO3) and TMAO is reduced to TMA by TMAO reductase. This figure has been adapted from the literature^2,6,7,9,11^.

The gut microbiome has recently been shown to play an important role in cardiovascular disease (CVD) and various other disorders^1–3^. Especially the gut metabolite trimethylamine-N-oxide (TMAO) has drawn a lot of attention, as it proved to be an important biomarker for CVD^1,4,5^. A schematic overview of the TMAO biosynthesis is depicted in Fig. 1. Wang *et al*. studied the influence of gut metabolites and the gut microbiome on CVD by comparing the metabolic profile between healthy volunteers and patients who had suffered a heart attack^6^. Not only TMAO, but also its precursor choline was found to be significantly correlated with an increased risk for CVD. In the following years, more research focused on elucidating the role of TMAO and the gut microbiome in CVD. The clinical relevance of TMAO was emphasized by a positive correlation between plasma levels of TMAO and major cardiovascular events (death, myocardial infarction, or stroke) in 4000 patients^3^. In addition to TMAO formation, choline is oxidized into betaine, which is mostly occurring in the renal cortex^5^. Betaine has also been reported as a biomarker for CVD^7^. L-carnitine, like choline and betaine, is a substrate for the gut microbiome to produce trimethylamine (TMA), a precursor of TMAO^2,8^. Koeth *et al*. showed that L-carnitine can also contribute to TMAO production via a distinct route. They demonstrated that the majority of dietary L-carnitine is converted to γ-butyrobetaine (GBB) instead of TMA^9^. GBB is converted into TMAO via TMA and accelerates atherosclerosis in mice. L-carnitine and choline are mostly derived from diet and are also endogenously synthesized^2,10^. These metabolites are the main source of the TMAO biosynthesis. L-carnitine, GBB, choline and betaine are all substrates for the gut microbiota to synthesize the precursor of TMAO: TMA^11^. Therefore, targeting these metabolites with the end product TMAO in a single analytical platform, can provide important information about the TMAO biosynthesis pathway in relation to cardiovascular diseases.

Techniques that have been used to analyze gut metabolites are proton nuclear magnetic resonance (^1^H-NMR) and mass spectrometry. There are only a few studies that analyzed TMAO with ^1^H-NMR^12,13^. This is probably due to the difficulties that are faced during ^1^H-NMR analysis of the TMAO metabolism, such as the inability to distinguish betaine signals from TMAO signals at pH values above 5.8^14^ and the overlap of glucose signals with TMAO, betaine and choline^15^. The latter is especially problematic for plasma since glucose is highly abundant in this matrix. Combined with the limited sensitivity of ^1^H-NMR, one can expect that mass spectrometry (MS) is the method of choice for the analysis of gut metabolites in biological fluids.

In order to increase throughput, flow injection analysis (FIA) and direct injection (DI) MS methods have been developed to measure gut metabolites^16,17^. Although the analysis time is minimized in these studies, ion suppression and sensitivity are not addressed in the method validation. This makes it difficult to assess to what extent the matrix is interfering during these analyses. Moreover, these methods were only used to measure urine. Therefore, it is questionable whether these methods are suitable for analyzing gut metabolites in plasma as this is a more complex matrix due to the presence of proteins and lipids^18–20^. Over the last five years, numerous analytical methods have been developed to study gut metabolites in plasma with liquid chromatography (LC)^2,3,5,21–29^. Both normal phase and reversed phase stationary phases have been used for the chromatographic separation. Fast hydrophilic interaction liquid chromatography (HILIC) analysis has been used to analyze TMA and TMAO in plasma in 2.5 minutes^26^. However, TMAO levels alone do not provide a complete picture to determine the increased risk for CVD after choline and L-carnitine ingestion. Recently, methods have been developed that included TMAO, betaine, choline and carnitine in their target analytes^24,28,29^. However, the throughput of the methods is compromised since the analysis time is 10 minutes or more. To our knowledge, no method has been published yet targeting the full TMAO biosynthesis pathway, as depicted in Fig. 1, in a high-throughput fashion.

In this study, a fast LC-ESI-MS/MS method was developed and validated for the analysis of the gut metabolites L-carnitine, GBB, choline and betaine and TMAO in plasma. An analytical column with a C18 stationary phase was used to realize an on-line cleanup of the sample before introducing it to the mass spectrometer. This stationary phase does not retain the analytes but does retain important matrix interferences, such as (phospho-)lipids. This type of chromatography is also referred to as negative chromatography^30^. As a result, a fast analysis of the gut metabolites in a cleaned flow-through is possible using ESI-MS/MS. This allowed us to quantify the five gut metabolites with an analysis time of only three minutes. A main evaluation point of the method is the influence of the matrix components on the ionization process and means to correct for matrix effect. This is especially important for high-throughput LC-MS since these platforms often have less separation, which generally results in a negative effect on electrospray ionization. To stress the importance of the on-line cleanup, we compared our LC-method to a FIA-MS method. The major contributors to ion suppression were identified by evaluating signal suppression of known ion suppressors from literature. We also evaluated the influence of the blood collection procedures on the gut metabolite concentrations. This should give insight into the performance of the method in different blood matrices and whether it is possible to compare different matrices to each other. The method was shown to be applicable to blood as is illustrated in a challenge test with a choline-containing beverage. Here, gut metabolite levels were analyzed in fasted serum levels and compared to serum levels taken about 30 minutes after the consumption of a standardized liquid meal.

## Results and Discussion

### Liquid chromatography-mass spectrometry

#### Method development

**Figure 2:**
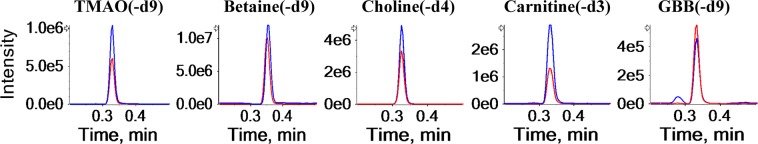
Extracted ion chromatograms of the LC-MS/MS analysis of the gut metabolites (blue line) and their deuterated internal standards (red line). There are a minimum of 12 data points on each peak. The data is obtained from a pooled serum sample. The chromatograms demonstrate a clear overlap in retention time and peak area of the deuterated internal standards.

In the first stage of the method development, unique precursor-product ion transmissions were optimized for all standards and deuterated internal standards. Betaine-d9 was chosen as a deuterated internal standard for betaine because we found background interferences in usable multiple reaction monitoring (MRM) transition for betaine-d3 in plasma. In the LC-MS analysis, the most important MS variable was the collision energy, which was optimized to reach the highest sensitivity. The LC gradient started with 95% water to maximize the retention of the matrix interferences by the LC column. Formic acid was added to facilitate the ionization of our target analytes. Since it was expected that matrix interference would mainly occur by the presence of lipids, a C18 column of 100 mm was used to provide sufficient load ability and retention of the matrix components. A shorter column would have improved the overall throughput slightly but could have also compromised the matrix removal. We did not use a time-consuming gradient because we did not aim for a chromatographic separation. Therefore, the mobile phase composition was almost directly switched to 100% organic solvent after the analysis of the analytes in order to wash the bound matrix components from the column before returning to the starting composition. The chromatograms demonstrated good peak shapes (Fig. 2).

Figure 2 demonstrates that the signal of the gut metabolites is in the same order of magnitude and the gut metabolites are coeluting with their deuterated internal standards, which is required for absolute quantification. An interesting finding was the appearance of an extra peak in the chromatogram of GBB. The extra peak elutes earlier than the larger peak and is baseline separated. Therefore, it did not cause any problems during peak integration. To ensure that this peak was another compound than GBB, we spiked a plasma sample with various concentrations of GBB (see supplementary information Fig. 1). The peak area of the earlier eluting peak did not change in size whilst the peak area of the later eluting peak did. This result indicates that the earlier eluting peak is indeed caused by another compound and not by the analyte GBB. The mobile phase of the FIA-MS method was optimized in order to reach to highest sensitivity. Therefore, 90% methanol and 0.1% formic acid were added to facilitate the electrospray ionization. Sensitivity is an important parameter in our method because we wanted to be able to measure a wide physiological range. This is especially important for TMAO, which is present in a wide physiological range (0.73–126 µM)^22^. Acetonitrile was used for the LC-MS analysis because of its stronger eluting power in comparison to methanol. This decreased the washing time of the LC column. The rest of the analysis parameters are the same for the FIA and LC analysis.

#### Matrix effect evaluation

Phospholipids are a major contributor to ion suppression^19,20^. Phosphatidylcholines (PCs) are the most abundant phospholipids and this particular type is responsible for most of the ion suppression caused by phospholipids because of their high abundance^31,32^. The physiological concentration of the sum of all PCs in human plasma mentioned in the literature is 211.3 mg/dL (157.0–327.0)^33–36^. For demonstration purposes in our study, one PC standard (211.3 mg/dL 1,2-dinonadecanoyl-sn-glycero-3-phosphocholine) was used to represent the PCs in plasma. The concentration of this standard was chosen to reflect the total concentration of PCs in plasma. Other well-known ion suppressors that are present in plasma in high concentrations are salts^37,38^. To explore the effect of salts on the ionization efficiency we used a physiological saline solution (154 mM NaCl).

**Figure 3:**
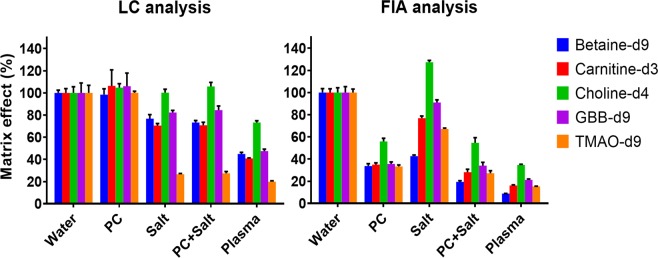
The matrix effect of various matrices on the peak area of the deuterated internal standards. Matrix effect was determined by the ratio of the peak area of the deuterated internal standards in a matrix sample to the peak area of the deuterated internal standards in a water sample. Physiological PC, salt, a combination of both and plasma have been evaluated for both FIA-MS and LC-MS.

In order to examine the performance of the on-line cleanup, the matrix effect of various added matrix components was evaluated for the LC-MS and a FIA-MS analysis. The matrix effect was determined by the ratio of the peak area of the deuterated internal standards in a matrix sample to the peak area of the deuterated internal standards in a water sample. We used deuterated internal standards because they are chemically similar to the corresponding gut metabolites and there were no background levels present in plasma for their MS/MS chromatograms. Figure 3 shows the matrix effect of the evaluated matrices on the peak area of the deuterated internal standards. The physiological PC concentration caused severe matrix effects when applying FIA-MS, reducing the signal to be 39 percent of the original signal. On the contrary, during the LC-MS analysis the PC matrix did not affect the signal. The matrix effect of the deuterated internal standards in the PC matrix was 103%, which was virtually identical to the reference values obtained in a water sample. This finding suggests that the LC column efficiently removed the PCs from the elution region of the analytes. The addition of a physiological salt concentration also caused substantial ion suppression. The FIA and LC analysis are both affected by this matrix because the salts coelute with the analytes in both methods. Betaine-d9 is considerably less affected during the LC analysis. This was expected since betaine has a slightly longer retention time on the LC column and will therefore elute further away from the unretained salts. Choline-d4 is not suppressed by the salt and is even enhanced in the FIA-MS method, which could be explained by the permanent positive net charge on the analyte. In the FIA, the PC and salt reduced the signal to 39 and 81% of the original signal, respectively, stressing the importance of PC removal before MS analysis. The matrix effect of PC and salt together already resembles the matrix effect caused by plasma, suggesting that these matrix constituents might be indeed responsible for the vast majority of matrix effect measured in plasma. This applies mainly to the PCs since these compounds are suppressing more than the salts. Although the suppression in the LC-MS analysis is considerably less, it has not been completely removed. Salts and other highly polar compounds (e.g. amino acids, creatinine, uric acid) that coelute with the gut metabolites still cause ion suppression. However, this remaining effect can be compensated by the use of deuterated internal standards. In addition to the reduced matrix effect, the elution of the retained matrix components can be switched to waste resulting in less contamination of the MS. In summary, the on-line matrix removal resulted in a cleaner analysis without suffering in terms of analysis time.

#### Method validation parameters

Table 1 summarizes the results of the method validation. In order to determine the LOD and LLOQ, a calibration curve was constructed in a water sample. Since we were aiming for absolute quantification using limited separation, we decided to calculate the LOD and LLOQ using the standard deviation of three replicates (see formula 3 and 4 in the methods section). This method comprises all sources of variability as well as the response. Lowest physiological concentration reported in the literature was at least six times higher than the LLOQ value, indicating a sufficient sensitivity of the developed method^22,39–41^. The correlation coefficient showed an excellent linearity (≥0.998) for all calibration curves. The analysis of blank samples resulted in a limited blank effect (<1.5%). The repeatability and intermediate precision of pooled citrated plasma samples demonstrated an RSD consistently below 10%, indicating a high repeatability of the analysis. The used gradient resulted in a robust analysis since we were able to analyze more than 5000 samples using the same column without a decrease in performance.

**Table 1:** Method validation parameters for the fast LC-ESI-MS/MS method.

Analyte	Elution time (min)	LOD (µM)	LLOQ (µM)	Linearity (R^2^)	Blank effect (%)	Repeatability (RSD, %)	Intermediate precision (RSD, %)
TMAO	0.33	0.04	0.1	0.999	0.5	6.1	5.2
Betaine	0.35	0.9	2.7	0.999	0.8	1.2	3.4
Choline	0.33	0.5	1.5	0.999	0.7	3.6	4.2
Carnitine	0.33	0.7	1.7	0.999	0.5	6.2	6.7
GBB	0.33	0.02	0.04	0.998	1.3	5.9	6.3

### Different blood collection procedures and freeze-thaw cycles

**Figure 4:**
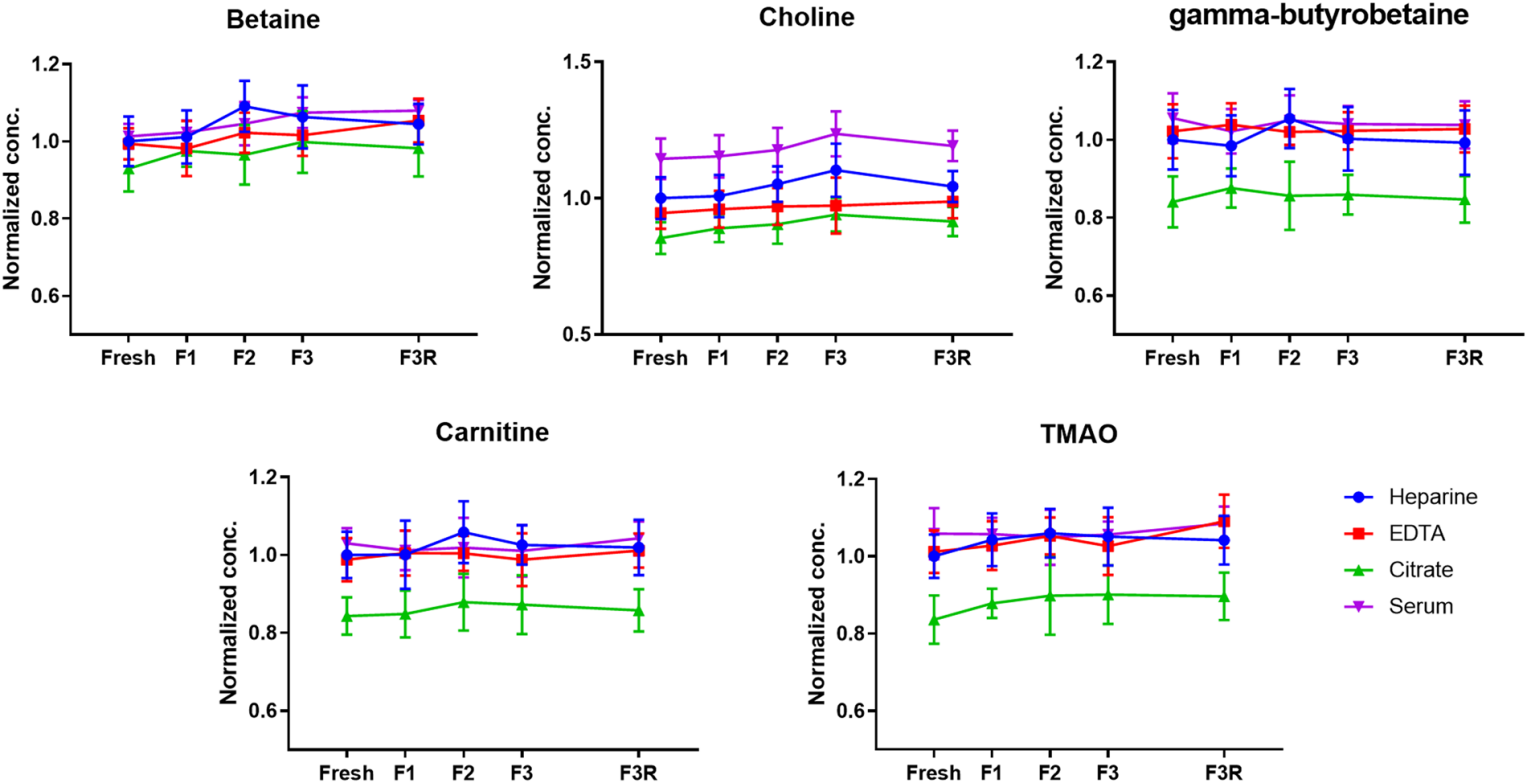
Different blood collection procedures of five healthy volunteers. Normalized concentrations of gut metabolites are plotted against the freeze-thaw (FT) cycles.

Differences in concentrations of small metabolites between different blood collection procedures have been observed and should be taken into account^42,43^. Therefore, we have evaluated four different blood collection procedures: ethylenediaminetetraacetic acid (EDTA) plasma, heparin plasma, citrated plasma and serum (Fig. 4). The validated analytical method showed a good linearity with a mean R^2^ of 0.998 (0.993–1.000) for all different collection procedures (Table 2) indicating the slopes of the calibration curves made in the different blood matrices as well as the calibration curve in a water sample were highly comparable with a mean RSD of 3.0% (1.6–3.4). The comparison to the uncorrected data (see Supplementary Information Table 1), which demonstrated a mean R^2^ of 0.971 (0.946–0.995) and a mean RSD of 28.9% (1.8–60.0), indicates that the deuterated internal standards are capable of extending the dynamic range, correcting the matrix effect of different blood matrices and allow for absolute quantification. We performed a one-way ANOVA and Post-Hoc analysis to investigate whether there was a statistical difference between the different collection procedures in terms of absolute concentrations. We have used freeze-thaw cycle 1 (FT1) for this comparison since most analyzed samples undergo one freeze-thaw cycle. All citrated plasma analyte concentrations demonstrated significantly lower values compared to the other blood collection procedures, except for betaine which did not reach this significance in comparison to EDTA and heparin treated blood. Lower citrate analyte concentrations can be explained by the way citrate plasma is obtained. When blood is treated with citrate, there is a dilution step involved because citrate is added as a liquid^42^. In our experiment, the blood to citrate ratio was 9:1 and on average, citrate analyte concentrations were 11% lower in comparison to EDTA and heparin analyte concentrations. This decrease clearly reflects the dilution ratio of liquid in citrate plasma collection tubes. In contrast to the other analytes, choline concentrations were 17% higher in serum samples in comparison to EDTA and heparin samples. A higher analyte concentration in serum compared to plasma is a well-known phenomenon in metabolomics and can be explained in two ways^43^. First of all, platelets release metabolites into serum during the coagulation process^44^. Secondly, the coagulation process causes the clotting of fibrinogen. By removing the clot, the volume fraction of coagulation proteins is removed. The remaining analytes are left in a lower volume which makes them more concentrated^45^. Since the last explanation should have increased all analyte concentrations in serum, it seems more likely that the platelets have released choline into the serum. This is in accordance with a study of Petty and Scrutton, who demonstrated that platelets store and release choline^46^. In order to demonstrate the effect of different freeze-thaw cycles, we have compared analyte levels of fresh blood samples to samples which were exposed to one or multiple freeze thaw-cycle(s). Figure 4 demonstrates that gut metabolite levels are not shown to be affected by the number of freeze-thaw cycles. This is advantageous because study samples often experience one or more freeze-thaw cycles. On the other hand, the type of whole blood treatments can have a significant impact on the measured gut metabolites concentrations. This finding emphasizes that the treatment of whole blood is an important parameter and that it should be taken into consideration during clinical applications.

**Table 2:** The slope and correlation coefficient of the calibration curves in a water sample and in different blood matrices using deuterated internal standard correction.

	TMAO		Betaine		Choline		Carnitine		GBB	
	Slope	R^2^	Slope	R^2^	Slope	R^2^	Slope	R^2^	Slope	R^2^
Water	0.20	0.998	0.031	0.999	0.11	0.999	0.052	0.998	0.71	0.998
Heparin	0.19	0.998	0.030	0.998	0.11	1.000	0.050	0.999	0.74	0.993
EDTA	0.19	0.993	0.033	0.999	0.12	0.999	0.054	0.999	0.73	0.997
Citrate	0.19	0.999	0.032	1.000	0.11	0.998	0.049	0.999	0.72	1.000
Serum	0.21	0.998	0.030	0.997	0.11	1.000	0.053	0.999	0.72	0.999
RSD of slopes (%)	3.1		3.4		3.2		3.2		1.6	

### Comparison of fasting state and meal challenge

**Figure 5:**
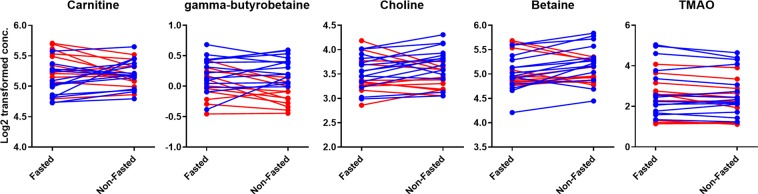
Before-after plots of gut metabolite serum levels of 25 volunteers during fasting and non-fasting (i.e. 33 min after a meal). The 14 male volunteers are depicted in blue and the 11 female volunteers are depicted in red.

**Figure 6:**
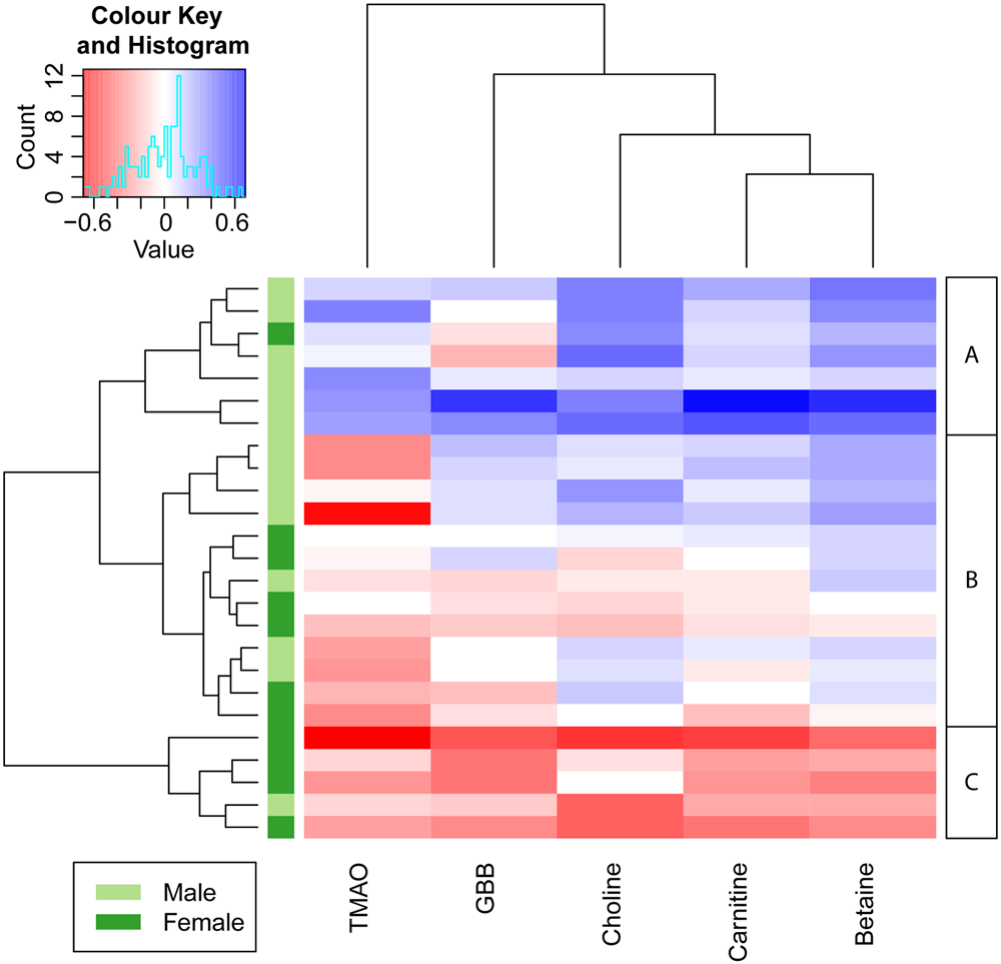
Heat map of the log2 transformed serum level differences between the fasted and non-fasted time point. Blue and red cells represent increased and decreased SLM-challenged metabolite levels in serum, respectively. The volunteers and metabolites are sorted by hierarchical clustering. Dendograms are used to show distances between samples (rows) and metabolites (columns). Based on the hierarchical clustering, three groups could be identified indicating volunteers with increased levels (**A**), limited differences (**B**) and decreased levels (**C**). It can be clearly seen that A and C consist of mainly male and female volunteers, respectively.

Figure 5 demonstrates the gut metabolite levels of 25 healthy volunteers during fasting (samples taken in the morning after overnight fasting) and half an hour after the consumption of a standardized liquid meal (SLM). The SLM contained 110 mg of choline – equivalent to the choline content of approximately one egg or 140 g of fish or meat^47^– and can therefore be considered as a substantial source of TMAO precursors. However, none of the targeted metabolites revealed a statistically significant difference between the fasting and SLM-challenged time point (FDR corrected p-value < 0.05, see Supplementary Information Table 2) and no clear trend could be identified in the before-after plots in Fig. 5, unless stratified by gender. The heat map in Fig. 6 also demonstrates that there is no uniform response to the SLM. Volunteers with increased (cluster A) and decreased (cluster C) gut metabolite levels after the SLM-challenge could clearly be distinguished based on hierarchical clustering. The difference in response might be explained by the gender of the volunteers since differences in betaine, carnitine, choline, γ-butyrobetaine serum levels were significantly correlated with the gender of the volunteers (FDR corrected p-value < 0.05, see Supplementary Information Table 3). In general, men and women demonstrated increased and decreased gut metabolite levels, respectively, after the challenge of SLM consumption compared to fasting. The decreasing levels in female volunteers were surprising because this trend has not been observed before after food intake. The observed trend does indicate that gender might be an important parameter in determining the gut metabolite response caused by food intake. However, when fasting and SLM-challenged serum levels were stratified by gender, only male betaine serum levels significantly changed between the two time points (FDR corrected p-value < 0.05, see Table 2 in the Supplementary Information). TMAO response was not correlated with the gender of the volunteers and showed most dissimilarities (distance in the dendrogram) in comparison to the other gut metabolites. The deviant behavior of TMAO might be explained by the reversibility of the TMA conversion to TMAO. Although a gender-related trend was observed, the differences between the two time points and volunteers might have also been caused by other sources of variability like the type and quantity of bacteria in the gut microbiome, variability of expression of choline dehydrogenase in the kidney cortex and biosynthesis of carnitine and choline. For example, a decrease in choline serum levels can be caused by an increased expression of choline dehydrogenase and an increase in carnitine and GBB could be a result of an increased biosynthesis of carnitine.

Although the gut metabolite serum levels did not change significantly between the fasted and SLM- challenged time point, an increase in glucose and branched-chain amino acids was demonstrated by Schutte *et al*. in the same sample collection and SLM-challenge as used in this study^48^. This indicates that the SLM is able to elevate certain metabolites in blood within half an hour. In addition to that, Lang *et al*. demonstrated that FMO3 catalyzes TMA *N*-oxygenation with a *k*_*cat*_ of more than thirty per minute indicating that TMAO could be formed within half an hour^49^. In contrast to our finding, Cho *et al*. found a significant increase in plasma TMAO levels after the consumption of foods containing TMAO or its precursors^50^. In that study, volunteers fasted overnight and were fed a diet containing eggs, beef or fish. After 15 minutes there was already a significant increase in plasma TMAO levels observed for all diets. The difference might be explained by the fact that only male volunteers were enrolled in the latter study. In accordance to our results, male volunteers demonstrated increased levels of gut metabolites after the consumption of a meal. Female volunteers, however, demonstrated an opposite trend in our results. Another difference can be found in the administration of ‘real’ dairy products in contrast to the SLM-challenge (‘artificial drink’) that has been used in our study. The SLM did not contain TMAO or carnitine and could therefore not increase TMAO levels by direct absorption of TMAO or via microbial conversion of carnitine. Other studies did not find a strong association between animal food intake and plasma concentrations of TMAO, betaine and choline^51,52^. Although clear differences in animal food intake could be identified, corresponding plasma levels of gut metabolites could not be correlated with this variation. However, the difference between fasting and food intake was not examined in these studies. Therefore, these studies do not reveal whether food consumption itself contributes to different gut metabolite levels.

## Conclusion

We have developed a high throughput LC-MS/MS platform to analyze gut metabolites in plasma and serum. The method can quantify five gut metabolites in three minutes per sample. By using an on-line cleanup approach, the most important ion suppressors, PCs, were removed from the elution region of the gut metabolites reducing the ion suppression significantly. The blood collection procedure had a significant impact on the measured concentrations of the gut metabolites. Therefore, the type of blood collection is of great importance within a study, and can be of great importance when different studies are compared to each other because inter-study variability could be caused by the collection procedures rather than, for instance, a diet or drug treatment. Our results did not demonstrate a significant diet-related effect of the gut metabolite levels in serum, since the gut metabolites hardly changed 30 minutes after the intake of an SLM. This is in contrast to another study, in which an increase was observed. However, this difference could have been caused by the administration of different types of food. We did observe volunteers with increased and decreased levels, which could have been driven by the gender. Therefore, the influence of fasting on the gut metabolite levels remains a subject of debate and is a parameter that should be studied in more detail. In summary, gut metabolites have been reported to have great potential to become a valuable biomarker in cardiovascular diseases, but this requires validation in large studies. Our simple and fast method facilitates further research and validation studies on the role of gut metabolites in relation to cardiovascular events and should stimulate other researchers to elucidate the biology behind this role. If TMAO is fully accepted as a valuable biomarker for cardiovascular diseases, our relatively easy method can help clinicians to make fast diagnoses of cardiovascular disease risks and can possibly contribute to prevent major cardiovascular events like death, myocardial infarction, or stroke.

## Methods

### Chemicals

Water was obtained from an arium pro UF/VF water purification system with a Sartopore 2 0.2 µm filter (Sartorius Stedim, Rotterdam, The Netherlands). High-performance liquid chromatography (HPLC) and ULC-MS grade methanol (MeOH) and HPLC grade acetonitrile (ACN) were purchased from Actu-all (Oss, The Netherlands). Formic acid 99% was purchased from Acros Organics (Geel, Belgium). 1,2-dinonadecanoyl-sn-glycero-3-phosphocholine (PC 19:0/19:0) was purchased from Avanti Polar Lipids, inc (Alabaster, USA). Sodium chloride originated from Sigma-Aldrich Chemie B.V. (Zwijndrecht, The Netherlands). The suppliers of the standards and deuterated internal standards can be found in the Supplementary Information (Table 4). Pooled citrated plasma (October 2016) was used for method development and was purchased from Sanquin (Amsterdam, The Netherlands).

### Sample preparation

A 10 µL aliquot of plasma was transferred into a 1.5-mL Eppendorf tube and mixed with 10 µL of deuterated internal standards in MeOH. During method validation, 10 µL of calibration standards in MeOH were added to the sample. A final volume of 100 µL was reached by adding MeOH. The Eppendorf tubes were thoroughly mixed by vortex mixing them for 30 seconds. Then, the Eppendorf tubes were centrifuged for 5 min (15700 g at 4 °C). After centrifugation, 80 µL of the supernatant was collected and transferred into an autosampler vial containing a 150 µL insert.

### LC-MS/MS

Samples were measured using a UPLC Agilent Infinity II (1290 Multisampler, 1290 Multicolumn Thermostat and 1290 High Speed Pump) (Agilent Technologies, Waldbronn, Germany) coupled to an AB SCIEX quadrupole-ion trap 6500 (QTRAP) (AB Sciex, Massachusetts, USA). The ionization source was the Turbo Spray Ion Drive and was set in positive mode with a capillary spray voltage set at 2500 V. Declustering potential, Entrance potential collision cell exit potential curtain gas collision gas temperature (TEM) ion source gas 1 and ion source gas 2 were set at 70 V, 10 V, 10 V, 20 psi, medium, 350 °C, 80 psi and 70 psi, respectively. The analyte and deuterated internal standard MRM transitions and the optimized collision energies are mentioned in the Supplementary Information (Table 4). We have developed an LC and FIA method. The LC analyses were carried out using an AccQ-Tag^TM^ Ultra C18 column (2.1 × 100 mm, 1.7 µm). The aqueous mobile phase A consisted of 0.1% formic acid in water and mobile phase B consisted of 0.1% formic acid in ACN. The gradient started at 5% B and was held at this value for 0.8 min. The gradient increased linearly to 50% B in 0.05 min and to 100% B in an extra 0.10 min. The mobile phase composition was held at 100% B for 1.25 min before it returned to 5% B in 0.02 min. Finally, the gradient was kept at 5% B for 0.78 min to re-equilibrate the column. The total analysis time was 3 min. FIA was carried out using a mobile phase consisting of formic acid/MeOH/H_2_O (0.1/90/10, v/v/v). The total analysis time was 1 minute. The injection volume was set at 1 µL and the flow rate at 700 µL/min for both LC and FIA analyses. The raw data was analyzed using MultiQuant (AB SCIEX, Version 3.0.2).

### Matrix effect evaluation

The matrix effect was determined by the ratio of the peak area of the deuterated internal standards in a plasma sample to the peak area of the deuterated internal standards in a water sample.

1 $$Matrix\,effect=\frac{Peak\,area\,ISTD\,in\,matrix}{Peak\,area\,ISTD\,in\,academic}\times 100 \% $$

In addition, individual matrix components were added to a water sample to determine their corresponding matrix effect on the target analytes: plasma, physiological salt, physiological phosphatidylcholine (PC) and the combination of physiological salt and PC. The physiological salt and PC solution consisted of 154 mM NaCl and 2,8 mM 1,2-dinonadecanoyl-sn-glycero-3-phosphocholine (PC 19:0/19:0), respectively.

### Calibration lines

The standards and their deuterated internal standards were weighed and dissolved in MeOH to reach a 1 mg/mL stock solution. The stock solutions were stored at −80 °C. A calibration line was constructed by several dilutions of the standard stock solutions. Eight calibration concentrations (C8-C1) in methanol were used for each standard in which C8 contained the highest concentration and the subsequent concentrations were 1:1 dilutions of the previous concentration. The ninth calibration point did not contain the standards (C0). The concentration of the deuterated internal standards was set to mimic the physiological concentrations of the corresponding analytes in plasma and was added to all nine calibration standards. The concentrations of C8 and the deuterated internal standards are mentioned in the Supplementary Information (Table 5). The calibration lines were constructed by plotting the ratio between the peak area of the standards and the peak area of their corresponding deuterated internal standards on the y-axis against the concentration of the analytes on the x-axis. Calibration lines were constructed in water samples and in plasma samples.

### Method validation parameters

Method validation was performed in water, plasma and serum. Repeatability (N = 5) was determined by the RSD of pooled citrated plasma samples. The intermediate precision (N = 26) was determined by the RSD of pooled citrated plasma samples measured on three different days. Blank effect was calculated by the ratio of peak area in blank samples (pure methanol, N = 4) to the peak area in a pooled serum samples (N = 14).

2 $$Blank\,effect=\frac{Peak\,area\,in\,blank\,samples\,}{Peak\,area\,in\,in\,pooled\,serum\,samples}\times 100 \% $$

Linearity, limit of detection (LOD) and lower limit of quantification (LLOQ) were determined using the calibration curve in water. The linearity of the calibration line was calculated by the correlation coefficient (R^2^). LOD (µM) and LLOQ (µM) were calculated using the standard deviation (SD) of the peak area of C1 (three replicates) and the peak area of a blank. The response factor (RF) was calculated by dividing the peak area of C1 by the [C1].

3 $$LOD=\frac{3\times SD(peak\,area\,C1)+peak\,area\,blank}{RF(\frac{peak\,area\,C1}{[C1]})}$$

4 $$LLOQ=\frac{10\times SD(peak\,area\,C1)+peak\,area\,blank}{RF(\frac{peak\,area\,C1}{[C1]})}$$

### Different blood collection procedures and freeze-thaw cycles

Fresh blood samples of five healthy volunteers were drawn. Every blood sample of each healthy volunteer was treated in four different ways within one hour. Plasma samples were prepared by collecting whole blood in EDTA, heparin or citrate containing tubes. Subsequently, tubes were centrifuged at 2000 g at 4 °C for 10 min. The supernatant was collected. Serum samples were prepared by allowing the whole blood to clot at room temperature for 30 min. Afterwards, the samples were centrifuged at 2000 g at 21 °C for 10 min. The supernatant was collected. For the TMAO platform, 10 μL of each sample (N = 3) was aliquoted immediately after plasma and serum were obtained. The aliquots were stored at −80 °C, except for the fresh sample measurements (fresh). A freeze-thaw (FT) cycle was defined by freezing the samples at −80 °C and having the samples thaw on crushed ice for 1 hour. Samples were stored at −80 °C for 1 day, 2 days and 3.5 hours for FT1, FT2 and FT3, respectively. After thawing, the samples were stored in the freezer or directly prepared and analyzed. The third freeze-thaw cycle was also thawed at room temperature (FT3R). The data was normalized on the average concentration of the fresh heparin treated plasma. Every compound for each healthy volunteer was normalized separately. The statistical analysis of the different blood collection procedures was performed by a one-way ANOVA analysis. For this, we used the data from the first freeze-thaw cycle, because a blood sample is commonly kept at −80 °C prior to analysis.

### Effect of a standardized liquid meal

The influence of a standardized liquid meal on the serum levels of the gut metabolites was evaluated in 25 older adults (11 women and 14 men with a mean age of 64.5 ± 5.2 years) participating in the Growing Old Together (GOTO) study^53^. Because the GOTO study only reports on the response to the GOTO lifestyle intervention study (baseline and follow-up), the challenge test with a standardized liquid meal (SLM) is described in this section. The first blood collection was in fasting status and took place between 8 and 9 am in the hospital after at least 10 h of fasting. The second blood collection was taken on average 33 min (SD ± 1 min) after a 200 mL SLM Nutridrink^TM^ challenge between 9 am and 12 am on the same day. Nutridrink is a liquid oral nutritional supplement (Nutricia Advanced Medical Nutrition, Zoetermeer, The Netherlands; 1.5 kcal/mL (6.25 kJ/mL), 35 En% fat, 49 En% carbohydrates, 16En% protein)^48^. The SLM contained 110 mg of choline and did not contain carnitine. The blood was collected in standard coagulation tubes of which serum was collected after coagulation of the blood. Quality control (QC) samples were prepared by pooling 5 µL of every individual study sample. In the batch design, a QC sample was analyzed after every tenth sample. Each QC sample and every seventh sample was measured twice. All tests were performed on the log2 transformed absolute concentrations of the gut metabolites. A paired t-test was used to assess differences between the two time points. The response to the challenge test was calculated as the difference of the log2 transformed non-fasted serum values and the log2 transformed absolute fasted serum values. These differences were tested for an association to gender using an unpaired, standard t-test. Further, a heat map of these differences was constructed using hierarchical clustering. A multiple testing correction (Benjamini-Hochberg, <0.05) was used to correct the p-values for the false discovery rate (FDR). The described studies were approved by the Medical Ethical Committee of the Leiden University Medical Center and comply with relevant guidelines and regulations. Volunteer inclusion was based on inform consent.

## Supplementary information

Supplementary information

## Data Availability

The authors are willing to provide further details upon request.

## References

[1] Wang Z (2011). Gut flora metabolism of phosphatidylcholine promotes cardiovascular disease. Nature.

[2] Koeth RA (2013). Intestinal microbiota metabolism of l-carnitine, a nutrient in red meat, promotes atherosclerosis. Nat. Med..

[3] Tang WHW (2013). Intestinal Microbial Metabolism of Phosphatidylcholine and Cardiovascular Risk. N. Engl. J. Med..

[4] Griffin JL, Wang X, Stanley E (2015). Does Our Gut Microbiome Predict Cardiovascular Risk?. Circ. Cardiovasc. Genet..

[5] Bennett BJ (2013). Trimethylamine-N-Oxide, a Metabolite Associated with Atherosclerosis, Exhibits Complex Genetic and Dietary Regulation. Cell Metab..

[6] Wang Z (2014). Prognostic value of choline and betaine depends on intestinal microbiota-generated metabolite trimethylamine-N-oxide. Eur. Heart J..

[7] Lever M, George PM, Dellow WJ, Scott RS, Chambers ST (2005). Homocysteine, glycine betaine, and N,N-dimethylglycine in patients attending a lipid clinic. Metabolism..

[8] Zeisel SH, Warrier M, Trimethylamine N (2017). -Oxide, the Microbiome, and Heart and Kidney Disease. Annu. Rev. Nutr..

[9] Koeth RA (2014). γ-butyrobetaine is a proatherogenic intermediate in gut microbial metabolism of L-carnitine to TMAO. Cell Metab..

[10] Zeisel SH, Da Costa KA (2009). Choline: An essential nutrient for public health. Nutr. Rev..

[11] Fennema D, Phillips IR, Shephard EA (2016). Trimethylamine and Trimethylamine N-Oxide, a Flavin-Containing Axis Implicated in Health and Disease. Drug Metab. Dispos..

[12] Wojtowicz W (2017). Serum and urine 1 H NMR-based metabolomics in the diagnosis of selected thyroid diseases. Sci. Rep..

[13] Garcia E (2017). NMR quantification of trimethylamine-N-oxide in human serum and plasma in the clinical laboratory setting. Clin. Biochem..

[14] Zuppi C (1997). 1H NMR spectra of normal urines: Reference ranges of the major metabolites. Clin. Chim. Acta.

[15] Nicholls AW, Mortishire-Smith RJ, Nicholson JK (2003). NMR Spectroscopic-Based Metabonomic Studies of Urinary Metabolite Variation in Acclimatizing Germ-Free Rats. Chem. Res. Toxicol..

[16] Hsu W-Y (2007). Rapid screening assay of trimethylaminuria in urine with matrix-assisted laser desorption/ionization time-of-flight mass spectrometry. Rapid Commun. Mass Spectrom..

[17] Johnson DW (2008). A flow injection electrospray ionization tandem mass spectrometric method for the simultaneous measurement of trimethylamine and trimethylamine N -oxide in urine. J. Mass Spectrom..

[18] Furey A, Moriarty M, Bane V, Kinsella B, Lehane M (2013). Ion suppression; A critical review on causes, evaluation, prevention and applications. Talanta.

[19] Ghosh C, Shinde CP, Chakraborty BS (2012). Influence of ionization source design on matrix effects during LC-ESI-MS/MS analysis. J. Chromatogr. B Anal. Technol. Biomed. Life Sci..

[20] Little JL, Wempe MF, Buchanan CM (2006). Liquid chromatography-mass spectrometry/mass spectrometry method development for drug metabolism studies: Examining lipid matrix ionization effects in plasma. J. Chromatogr. B Anal. Technol. Biomed. Life Sci..

[21] Kuka J (2014). Suppression of intestinal microbiota-dependent production of pro-atherogenic trimethylamine N-oxide by shifting L-carnitine microbial degradation. Life Sci..

[22] Wang Z (2014). Measurement of trimethylamine-N-oxide by stable isotope dilution liquid chromatography tandem mass spectrometry. Anal. Biochem..

[23] Zhao X, Zeisel SH, Zhang S (2015). Rapid LC-MRM-MS assay for simultaneous quantification of choline, betaine, trimethylamine, trimethylamine N -oxide, and creatinine in human plasma and urine. Electrophoresis.

[24] Steuer C, Schutz P, Bernasconi L, Huber AR (2016). Simultaneous determination of phosphatidylcholine-derived quaternary ammonium compounds by a LC-MS/MS method in human blood plasma, serum and urine samples. J. Chromatogr. B Anal. Technol. Biomed. Life Sci..

[25] Mi S, Zhao Y-Y, Jacobs RL, Curtis JM (2017). Simultaneous determination of trimethylamine and trimethylamine *N* -oxide in mouse plasma samples by hydrophilic interaction liquid chromatography coupled to tandem mass spectrometry. J. Sep. Sci..

[26] Heaney LM, Jones DJL, Mbasu RJ, Ng LL, Suzuki T (2016). High mass accuracy assay for trimethylamine N-oxide using stable-isotope dilution with liquid chromatography coupled to orthogonal acceleration time of flight mass spectrometry with multiple reaction monitoring. Anal. Bioanal. Chem..

[27] Ocque AJ, Stubbs JR, Nolin TD (2015). Development and validation of a simple UHPLC-MS/MS method for the simultaneous determination of trimethylamine N-oxide, choline, and betaine in human plasma and urine. J. Pharm. Biomed. Anal..

[28] Liu J (2016). Simultaneous targeted analysis of trimethylamine-N-oxide, choline, betaine, and carnitine by high performance liquid chromatography tandem mass spectrometry. J. Chromatogr. B Anal. Technol. Biomed. Life Sci..

[29] Kadar H (2016). A multiplexed targeted assay for high-throughput quantitative analysis of serum methylamines by ultra performance liquid chromatography coupled to high resolution mass spectrometry. Arch. Biochem. Biophys..

[30] Lee MFX, Chan ES, Tey BT (2014). Negative chromatography: Progress, applications and future perspectives. Process Biochem..

[31] Ismaiel OA, Zhang T, Jenkins RG, Karnes HT (2010). Investigation of endogenous blood plasma phospholipids, cholesterol and glycerides that contribute to matrix effects in bioanalysis by liquid chromatography/mass spectrometry. J. Chromatogr. B Anal. Technol. Biomed. Life Sci..

[32] Myher JJ, Kuksis A, Pind S (1989). Molecular species of glycerophospholipids and sphingomyelins of human erythrocytes: improved method of analysis. Lipids.

[33] Seppänen-Laakso T (2001). Major human plasma lipid classes determined by quantitative high-performance liquid chromatography, their variation and associations with phospholipid fatty acids. J. Chromatogr. B Biomed. Sci. Appl..

[34] Quehenberger O (2010). Lipidomics reveals a remarkable diversity of lipids in human plasma. J. Lipid Res..

[35] Dougherty RM, Galli C, Ferro-Luzzi A, Iacono JM (1987). Lipid and phospholipid fatty acid composition of plasma, red blood cells, and platelets and how they are affected by dietary lipids: a study of normal subjects from Italy, Finland, and the USA. Am. J. Clin. Nutr..

[36] Maldonado EN, Romero JR, Ochoa B, Aveldaño MI (2001). Lipid and fatty acid composition of canine lipoproteins. Comp. Biochem. Physiol. B. Biochem. Mol. Biol..

[37] Trufelli H, Palma P, Famiglini G, Cappiello G (2011). An overview of matrix effects in liquid chromatography-mass spectrometry. Indian J. Exp. Biol..

[38] Annesley TM (2003). Ion suppression in mass spectrometry. Clin. Chem..

[39] Bain MA, Faull R, Fornasini G, Milne RW, Evans AM (2006). Accumulation of trimethylamine and trimethylamine-N-oxide in end-stage renal disease patients undergoing haemodialysis. Nephrol. Dial. Transplant..

[40] Psychogios, N. *et al*. The human serum metabolome. *PLoS One* **6** (2011). 10.1371/journal.pone.0016957 PMC3040193 21359215

[41] Vernez L, Wenk M, Krähenbühl S (2004). Determination of carnitine and acylcarnitines in plasma by high-performance liquid chromatography/electrospray ionization ion trap tandem mass spectrometry. Rapid Commun. Mass Spectrom..

[42] Gonzalez-Covarrubias V, Dane A, Hankemeier T, Vreeken RJ (2013). The influence of citrate, EDTA, and heparin anticoagulants to human plasma LC-MS lipidomic profiling. Metabolomics.

[43] Yu Z (2011). Differences between human plasma and serum metabolite profiles. PLoS One.

[44] Naoki Y (1997). Sphingosine 1-phosphate, a bioactive sphingolipid abundantly stored in platelets, is a normal constituent of human plasma and serum. J. Biochem..

[45] Kronenberg F (1998). Influence of hematocrit on the measurement of lipoproteins demonstrated by the example of lipoprotein(a). Kidney Int..

[46] Petty AC, Scrutton MC (1993). Release of Choline Metabolites from Human Platelets: Evidence for Activation of Phospholipase D and of Phosphatidylcholine-specific Phospholipase C. Platelets.

[47] Patterson, K. Y. *et al*. USDA Database for the Choline Content of Common Foods In collaboration with. *Environ. Heal*. 1–37, 10.15482/USDA.ADC/1178141 (2008).

[48] Schutte BAM (2016). The effect of standardized food intake on the association between BMI and 1 H-NMR metabolites. Sci. Rep..

[49] Lang DH (1998). Isoform specificity of trimethylamine N-oxygenation by human flavin-containing monooxygenase (FMO) and P450 enzymes Selective catalysis by fmo3. Biochem. Pharmacol..

[50] Cho CE (2017). Trimethylamine-N-oxide (TMAO) response to animal source foods varies among healthy young men and is influenced by their gut microbiota composition: A randomized controlled trial. Mol. Nutr. Food Res..

[51] Kühn T (2017). Intra-individual variation of plasma trimethylamine-N-oxide (TMAO), betaine and choline over 1 year. Clin. Chem. Lab. Med..

[52] Rohrmann S, Linseisen J, Allenspach M, von Eckardstein A, Mu ller D (2016). Plasma Concentrations of Trimethylamine-N-oxide Are Directly Associated with Dairy Food Consumption and Low-Grade Inflammation in a German Adult Population. J. Nutr..

[53] van de Rest O (2016). Metabolic effects of a 13-weeks lifestyle intervention in older adults: The Growing Old Together Study. Aging (Albany. NY)..

