# Supplementary material for: Fast LC-ESI-MS/MS analysis and influence of sampling conditions for gut metabolites in plasma and serum
Source: Sci Rep. 2019 Aug 26;9:12370. doi: 10.1038/s41598-019-48876-w (PMC6710273; doi:10.1038/s41598-019-48876-w)
Supplement: Supplementary file 1 — Supplementary information [file 41598_2019_48876_MOESM1_ESM.docx]

**Fast LC-ESI-MS/MS analysis and influence of sampling conditions for gut metabolites in plasma and serum**

**Tom van der Laan, Tim Kloots, Marian Beekman, Alida Kindt, Anne-Charlotte Dubbelman, Amy Harms, Cornelia M van Duijn, P. Eline Slagboom, Thomas Hankemeier**

# Supplementary information

| 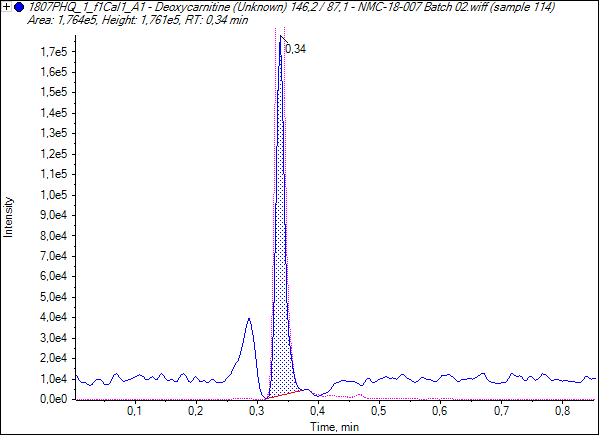C0 | 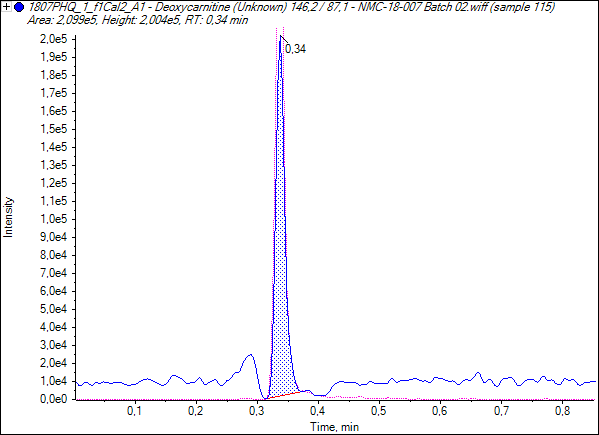C1 | 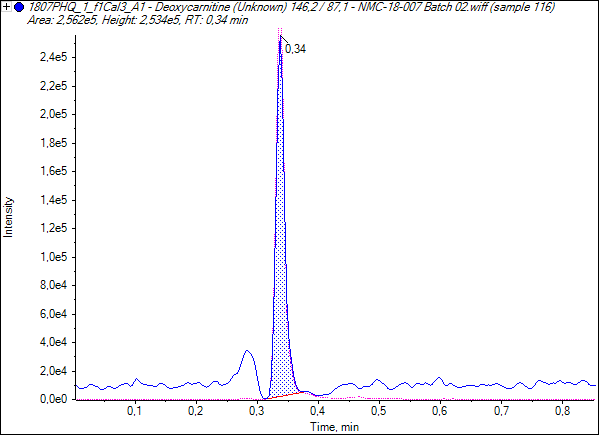C2 |
| --- | --- | --- |
| 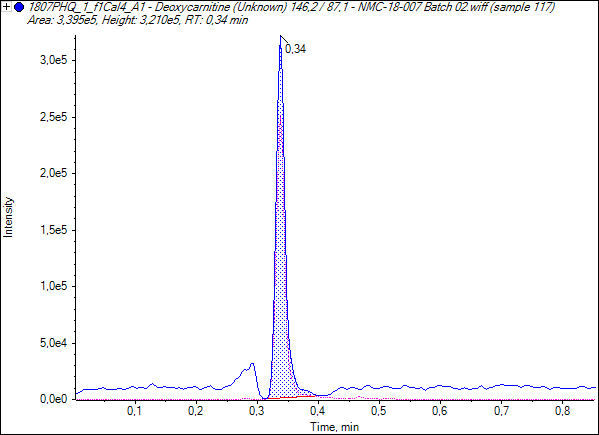C3 | 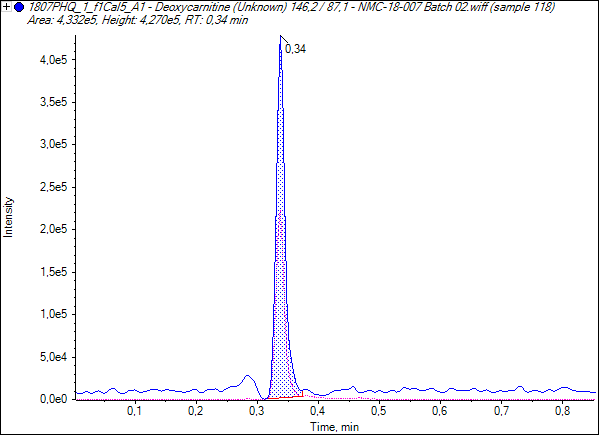C4 | 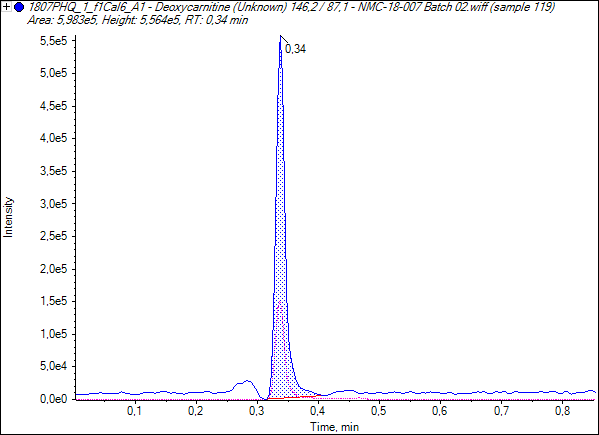C5 |
| 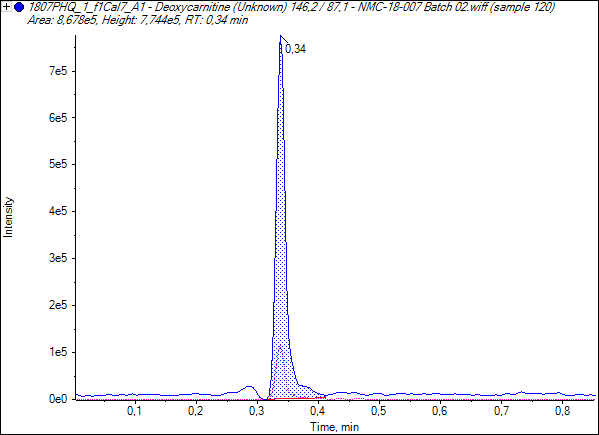C6 | 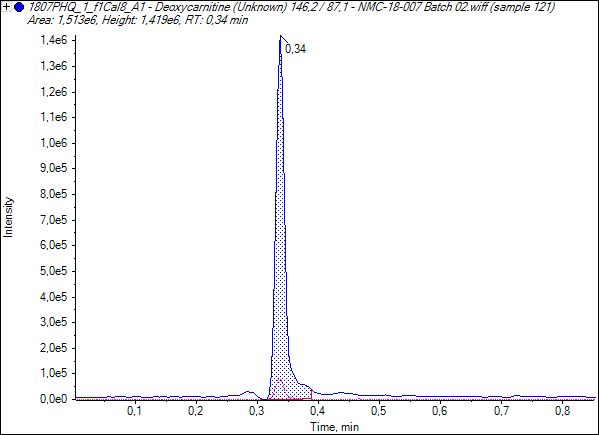C7 |  |

**Figure 1.** The extracted ion chromatogram of γ-butyrobetaine at increasing concentrations (C0-7) of the standard. The blue line represents γ-butyrobetaine signal and the red line represents the deuterated internal standard γ-butyrobetaine-d9 signal. It can be clearly seen that the small peak before γ-butyrobetaine is baseline separated and is not increasing with increasing concentrations of the standard γ-butyrobetaine.

**Table 1. The slope and correlation coefficient of the calibration curves in a water sample and in different blood matrices without using deuterated internal standard correction.**

|  | TMAO | |  | Betaine | |  | Choline | |  | Carnitine | |  | GBB | |
| --- | --- | --- | --- | --- | --- | --- | --- | --- | --- | --- | --- | --- | --- | --- |
|  | Slope | R^2^ |  | Slope | R^2^ |  | Slope | R^2^ |  | Slope | R^2^ |  | Slope | R^2^ |
| Water | 97523 | 0.950 |  | 40692 | 0.978 |  | 77780 | 0.960 |  | 142427 | 0.973 |  | 128063 | 0.956 |
| Heparin | 72464 | 0.983 |  | 13422 | 0.981 |  | 79004 | 0.966 |  | 82676 | 0.966 |  | 45480 | 0.986 |
| EDTA | 74382 | 0.975 |  | 11887 | 0.977 |  | 80663 | 0.946 |  | 69215 | 0.946 |  | 36374 | 0.962 |
| Citrate | 81862 | 0.988 |  | 12458 | 0.988 |  | 81660 | 0.951 |  | 72620 | 0.951 |  | 42873 | 0.995 |
| Serum | 71976 | 0.985 |  | 14119 | 0.988 |  | 80952 | 0.970 |  | 81514 | 0.970 |  | 46438 | 0.990 |
| RSD of slopes (%) | 12.1 |  |  | 60.0 |  |  | 1.8 |  |  | 29.9 |  |  | 57.3 |  |

**Table 2: Statistical difference between the fasting and non-fasting time point for all volunteers and stratified by gender. Significant differences are indicated (* indicates p-value <0.05).**

| **Compound name** | **gender** | **p-value** | **FDR corrected** |
| --- | --- | --- | --- |
| **Betaine** | all | 0.076 | 0.142 |
| **Carnitine** | all | 0.849 | 0.849 |
| **Choline** | all | 0.296 | 0.370 |
| **gamma-butyrobetaine** | all | 0.441 | 0.508 |
| **TMAO** | all | 0.056 | 0.120 |
| **Betaine** | Male | 0.001* | 0.009* |
| **Carnitine** | Male | 0.036* | 0.091 |
| **Choline** | Male | 0.013* | 0.065 |
| **gamma-butyrobetaine** | Male | 0.141 | 0.235 |
| **TMAO** | Male | 0.544 | 0.583 |
| **Betaine** | Female | 0.243 | 0.349 |
| **Carnitine** | Female | 0.031* | 0.091 |
| **Choline** | Female | 0.256 | 0.349 |
| **gamma-butyrobetaine** | Female | 0.007* | 0.053 |
| **TMAO** | Female | 0.020* | 0.075 |

**Table 3: Association between gender and log2 transformed fasted and non-fasted serum level differences. Significant correlations are indicated (* indicates p-value <0.05).**

| **Compound name** | **p-value** | **FDR corrected** |
| --- | --- | --- |
| **Betaine** | 0.001* | 0.004* |
| **Carnitine** | 0.002* | 0.004* |
| **Choline** | 0.013* | 0.016* |
| **gamma-butyrobetaine** | 0.002* | 0.004* |
| **TMAO** | 0.211 | 0.211 |

**Table 4: Supplier, collision energy and transitions of the standards and deuterated internal standards**

| Name | (Deuterated internal) standard | Supplier | Collision energy (eV) | Transition (m/z) |
| --- | --- | --- | --- | --- |
| Betaine | Betaine hydrochloride | Sigma Aldrich | 30 | 118.1🡪58.2 |
| Betaine d-9 | N-(Carboxymethyl)-trimethyl-d9 ammonium chloride | CDN isotopes | 30 | 127.1 🡪 66.1 |
| L-carnitine | L-carnitine hydrochloride | Sigma Aldrich | 25 | 162.3🡪85.1 |
| L-carnitine-d3 | L-carnitine-d3 hydrochloride | CDN isotopes | 25 | 165.3🡪85.1 |
| choline | Choline chloride | Sigma Aldrich | 25 | 104.2🡪60.1 |
| choline-d4 | Choline-1,1,2,2-d4 chloride | CDN isotopes | 25 | 108.2🡪60.1 |
| TMAO | Trimethylamine N-oxide dihydrate | Sigma Aldrich | 25 | 76.1🡪58.1 |
| TMAO-d9 | Trimethylamine N-oxide-d9 | Cambridge Isotope Laboratories | 25 | 85.1🡪66.1 |
| GBB | (3-carboxypropyl)trimethyl-ammonium chloride | Sigma Aldrich | 20 | 146.2🡪87.1 |
| GBB-d9 | (3-carboxypropyl)trimethyl-d9-ammonium chloride | CDN isotopes | 20 | 155.2🡪87.1 |

**Table 5: Concentration of the C8 of the standards and deuterated internal standards.**

| C8 concentration of the standards | |  | Deuterated internal standard concentration | | |  |
| --- | --- | --- | --- | --- | --- | --- |
| Name | concentration (µM) |  | | Name | concentration (µM) | |
| TMAO | 43.2 |  |  | TMAO-d9 | 3.56 | |
| Betaine | 716.1 |  |  | Betaine-d9 | 24.59 | |
| Choline | 286.5 |  |  | Choline-d4 | 13.92 | |
| Carnitine | 556.5 |  |  | Carnitine-d3 | 19.93 | |
| γ-butyrobetaine | 17.6 |  |  | γ-butyrobetaine -d9 | 1.05 | |
